# Prescription Drug Labeling Medication Errors: A Big Deal for Pharmacists

**DOI:** 10.4103/0975-1483.62218

**Published:** 2010

**Authors:** G Jeetu, T Girish

**Affiliations:** *Department of Pharmacy Practice, Manipal College of Pharmaceutical Sciences, Manipal University, Manipal, Karnataka, India*

**Keywords:** Health care profession, labeling, medication error

## Abstract

Today, in the health care profession, all types of medication errors including missed dose, wrong dosage forms, wrong time interval, wrong route, etc., are a big deal for better patient care. Today, problems related to medications are common in the healthcare profession, and are responsible for significant morbidity, mortality, and cost. Several recent studies have demonstrated that patients frequently have difficulty in reading and understanding medication labels. According to the Institute of Medicine report, “Preventing Medication Errors”, cited poor labeling as a central cause for medication errors in the USA. Evidence suggests that specific content and format of prescription drug labels facilitate communication with and comprehension by patients. Efforts to improve the labels should be guided by such evidence, although an additional study assessing the influence of label design on medication-taking behavior and health outcomes is needed. Several policy options exist to require minimal standards to optimize medical therapy, particularly in light of the new Medicare prescription drug benefit.

## INTRODUCTION

In this world, errors due to look-alike or sound-alike medication names are common and are responsible for thousands of deaths and millions of dollars in cost every year. A study has shown that up to 25% of all medication errors are attributed to name confusion and 33% to packaging and labeling confusion. Thousands of medication name pairs have been confused based on similar appearances or sounds when written or spoken or have been identified as having the potential for confusion.[1] According to report of the Institute of Medicine of the National Academies, “Preventing Medication Errors,” approximately 1.5 million preventable adverse drug events occur each year. However, more than one-third of adverse drug events take place in outpatient settings at a cost approaching $1 billion annually.[2] It has been expected that a large share of outpatient medication errors occur as a result of patients themselves not administering a medicine as intended.[3]

## NEED OF ATTENTION

According to the Institute of Medicine (IOM) report, cited poor labeling as a central cause for medication errors.[4] Attention to the origin causes of medication errors leading to adverse events has most often been attributed to the providers and health care systems contributing character to errors during the prescribing, ordering, dispensing, or administering of a medicine.[5,6] The reason for focusing on those causes may be that most studies investigating medication errors have been conducted in inpatient hospitals or nursing homes.[7] Studies have shown that variability in drug labeling and the use of certain terminology can adversely affect a patient’s understanding of medication instructions.[8,9] The Joint Commission and the National Coordinating Council for Medication Error Reporting and Prevention (NCC MERP) have provided guidance for physicians on how to write “Sig.” messages, with recommendations to avoid certain wording, acronyms, and Latin phrases that have been linked to medication errors.[10,11] Chronically sick patients and the elderly are at the greatest risk for experiencing medication errors as they take more prescription drugs per annum than younger and healthier patients, and visual or cognitive impairments by age may limit reading ease and comprehension.[12–16] Patients growingly self-manage greater numbers of prescriptions and over-the-counter medications. The jeopardy for miscommunication and error may be supplementally compounded since the average older adult sees several different health care providers annually.[17]

## VARIABILITY AND QUALITY OF MEDICATION CONTAINER LABELS

Medication errors occur regularly and poor medication labeling is cited as a potential cause. The experts studied and assessed the format, content, and variability of prescription drug container labels dispensed in a population. A study has been done by William H *et al*. and they evaluated 85 labels after excluding 11-Ibuprofen prescriptions that were filled with over-the counter containers that lacked labels printed at the pharmacy. The pharmacy name or logo was the most prominent item on 71 (84%) of the labels, with a mean font size of 13.6 point. Font sizes were lesser for medication instructions (9.3 point), medication name (8.9 point), and warning and instruction stickers (6.5 point). Color, boldfacing, and highlighting were most often used to identify the pharmacy and items most useful to pharmacists. While the content of the main label was generally consistent, there was substantial variability in the content of instruction and warning stickers from different pharmacies, and independent pharmacies were less likely to use such stickers. None of the Ibuprofen containers were delivered with Food and Drug Administration-approved medication guides, as required by law. After this study, they concluded that the format of most container labels emphasizes pharmacy characteristics and items frequently used by pharmacists rather than use instructions or medication warnings. The content of warning and instruction stickers is highly variable depending on the pharmacy selected.[18]

## PHYSICIAN-PATIENT COMMUNICATION

Most of the physicians with official responsibilities to convey instructions on proper medication use have frequently been found to be ineffective in their role. Research has shown that physicians frequently miss opportunities to counsel their patients on how to self-administer their medicines.[19,20] Health literacy studies have also highlighted that many physicians do not communicate health and treatment information in a manner that can be understood by patients with limited literacy skills.[21] If the patient leaves the physician office without the information needed to correctly implement the prescribed regimen, the pharmacist, at the point of dispensing medicines, would be next in line to counsel patients. Studies have shown that pharmacists also often fail to orally communicate detailed information to patients to support their adherence with prescribed regimens.[22,23] The last opportunity for counseling is the container label and accompanying print materials such as container label, patient package inserts, consumer medication information, medication guides, etc., which have been found to be long, complex, and written at a level too difficult for a majority of patients, regardless of their literacy level, to comprehend and use.[24,25]

## HEALTH LITERACY AND MEDICAL SAFETY

Several studies have found limited health literacy to be significantly associated with a poorer understanding of medication names, indications, and instructions.[26–28] Recently, health literacy was specifically well known within a seminal report released by the National Council for Patient Information and Education (NCPIE).[29] The report refers to health literacy as a national concern with regard to a patient’s understanding, safe use, and proper adherence to medication regimens.[29] A multi site study conducted by Davis *et al*. and they showed that among adults receiving primary care at community health centers, there was a high prevalence of patients, especially those with limited literacy, misunderstanding apparently simple dose instructions provided on the primary label of medication containers.[30] In this study, 46% of adults misunderstood at least one prescription container label they encountered. The problem extends to the auxiliary sticker labels that provide accompanying warnings and directions for use of the medicine.[29,31] Other studies demonstrated that over half (53%) of patients, especially those with limited literacy, had difficulty interpreting text and icons usually used on auxiliary warning instructions.[29]

## PARTICIPANTS OF THIS ERROR SYSTEM

The problems associated with prescription container labeling are ultimately the result of an apparent lack of standards and regulatory errors. This is a matter of patient safety and successful therapeutic outcomes. A lack of integration among the existing health information systems that support a rising number of prescribers and the majority of dispensing pharmacies also add to labeling difficulties.[31]

### The prescriber

The container label offers perhaps the only written documentation of dosage or usage directions for the patient, which is imparted through the physicians’ prescription. In most pharmacies today, whatever the physician writes is what is transcribed onto the container label. Although there may be a finite number of ways a prescription can be written, the same dose and frequency schedule for a prescribed drug may be written in several different ways. Today most of the physicians also use a range of Latin abbreviations to identify drug dose and frequency, rendering the prescription uninterruptable to most patients. This becomes especially challenging as many patients, especially the elderly, may have more than one health care provider prescribing medicines. It is unclear if physicians and other prescribing health care providers receive adequate training in writing prescriptions. Although electronic prescribing offers options for enhanced safety, it is still necessary to determine what physician prescribing notations optimize patient’s safe and effective use of medications.[32]

### The dispensing pharmacy

In a modern study, conducted by Shrank *et al*. and they showed after gathered data from identically written prescriptions filled for four commonly prescribed drugs (Atorvastatin, Alendronate, Trimethoprim-Sulfamethoxazole, and Ibuprofen) in six different pharmacies (two chains, two independent, and two grocery stores) in four diverse cities.[33] The evaluation of the format of labels on filled prescriptions suggested that labels were not designed to optimize patient understanding of medication administration instructions or warnings. The largest item on nearly all of the labels was the pharmacy logo. The label items that were emphasized were useful to identify the pharmacy and to enhance the practice of the pharmacist, but not to help patients to safely and appropriately administer medication. In the reported study, between 8% and 25% of containers did not include any warning or instruction stickers. Among the 85 labels evaluated, dose frequency was omitted on 6% of instructions.[34] A total of 27% of the translated instructions had a lexile reading grade level above a high school level.[31]

## HEALTH INFORMATION TECHNOLOGY: CAN IT REDUCE THIS MEDICATION ERROR

To compare handwritten and electronically generated prescription drug instructions, by Stephen *et al*. conducted a study and they assessed the variability of medication instructions and their compliance with Joint Commission and NCC MERP recommendations at the point of prescribing three medications with the common dosage instruction, “Take one tablet a day.” And they compared 85 handwritten prescriptions from a hospital in the Southeast with 1326 electronically generated prescriptions from an academic practice in the Midwest. The majority (61%) of handwritten prescriptions did not adhere to Joint Commission and NCC MERP recommendations to avoid Latin phrases. In contrast, only 1% of electronically generated prescriptions contained Latin abbreviations. Electronically generated prescriptions also had less variability than handwritten prescriptions. Most electronically generated medication instructions (93%) were default Sig. messages, which automatically appear within the text box in the electronic health record (EHR). A recent 2008 IOM report has provided clear evidence to support best practices for drug labeling. The use of EHRs offers an opportunity to adopt these practices and reduce instruction variability and the use of Latin terminology. Efforts should be taken to set standards for the writing of Sig. messages to promote patient safety and improve patient understanding of medication instructions, thereby reducing the number of preventable adverse drug events.[35,36]

## NEED TO IMPROVE THIS MEDICATION ERROR

There is evidence available to detail “best practices” for improving dosage or usage instructions written by the prescribing physician and the format and content of prescription medication container labels designed by the dispensing pharmacy.[37] The use of standard and more explicit dosage or usage instructions can improve patients’ functional understanding of how and when to take a medicine.[30] Evidences are available for best practices in labeling format and content, such as increasing font size, using clear and simple language, using headers, and placing a more appropriate emphasis on organizing label content around what is most important for patients such as drug name, dose, dosage or usage instructions, patient name, doctor name, quantity, refill information, and provider content such as pharmacy name, logo and national drug code number should be in optimal font size. A complete list of evidence-based, recommended standards for format, content, and instruction is as follows:[37]

1. Use explicit text to describe dosage and interval in instructions.
2. Use a universal medication schedule (UMS) to convey and simplify dosage and use instructions.
3. Organize labels in a patient-centered manner.
4. According to need, include indication for use.
5. Simplify language, avoiding unfamiliar words or medical jargon.
6. Improve typography, use larger, sans serif font.
7. When applicable, use numeric versus alphabet characters.
8. Use typographic cues (bolding and highlighting) for patient content only.
9. Use horizontal text only.
10. Use a standard icon system for signaling and organizing auxiliary warnings and instructions.

## CONCLUSION

A complete review of the published literature to evaluate the data regarding the best possible content and format of prescription labels might improve readability, understanding, and medication use. The evidence suggests that patients request information about a medication’s indication, expected benefits, duration of therapy, and a meticulous list of potential adverse effects. The evidence about label format supports the use of larger fonts, lists, headers, and white space, using simple language and logical organization to improve readability and comprehension. Patients must be able to easily realize how to use prescription drugs correctly. Standardizing and integrating drug labeling must be a central goal to ensure that best practices are implemented because evidence is already available to target improvements. This should be viewed as a short-term goal for policymakers, and some states have already made evolution to this end. In the long term, additional challenges for drug labeling include efforts to seek labeling concordance in other languages because not all prescription drug information and directions are presently available to non-English speakers. A formative response to labeling troubles would also extend to addressing how health care providers communicate to patients the information that is required to safely administer prescribed medicines. Mostly, health technology used by an increasing number of providers at the point of writing the prescription should be incorporated with the software used by dispensing pharmacies to fill it and print out the labeling components. This would provide another layer of quality assurance that could reduce variability and the risk that directions become lost in conversion.
